# Gewichtsverluste und Mangelernährungsrisiko bei geriatrischen PatientInnen

**DOI:** 10.1007/s00391-021-01900-z

**Published:** 2021-05-05

**Authors:** Fabian Graeb, Reinhold Wolke, Petra Reiber

**Affiliations:** Fakultät Soziale Arbeit, Gesundheit und Pflege, Institut für Gesundheits- und Pflegewissenschaften, Hochschule Esslingen – University of Applied Sciences, Flandernstraße 101, 73732 Esslingen, Deutschland

**Keywords:** Routinedaten, Prävention, Krankenhaus, Mangelernährungsscreening, Morbidität, Prevention, Routine data, Hospital, Malnutrition screening, Morbidity

## Abstract

**Hintergrund:**

Mangelernährung stellt eine große Herausforderung im klinischen Alltag dar und ist mit einer erhöhten Mortalität assoziiert.

**Ziel der Arbeit:**

Im vom Bundesministerium für Bildung und Forschung (BMBF) geförderten Forschungsprojekt *Prävention und Behandlung von Mangelernährung bei geriatrischen Patienten im Krankenhaus* werden Routinedaten ausgewertet. Ziel ist es, die Ursachen eines in der Klinik erworbenen Mangelernährungsrisikos aufzudecken.

**Material und Methoden:**

Ausgewertet werden anonymisierte Daten von insgesamt 2058 PflegeheimbewohnerInnen aus 19 Pflegeeinrichtungen mit mindestens 3‑tägigem Klinikaufenthalt. Mangelernährungsrisiko wird mithilfe der kombinierten Screenings MUST/PEMU (Malnutrition Universal Screnning Tool/Pflegerische Erfassung von Mangelernährung und deren Ursachen) , die manifeste Mangelernährung entsprechend den ESPEN-Kriterien (European Society for Clinical Nutrition and Metabolism) bestimmt.

**Ergebnisse:**

Ein initialer Anteil von 36,2 % (*n* = 744) weist ein Mangelernährungsrisiko und 12,7 % (*n* = 262) eine manifeste Mangelernährung auf; die Anteile steigen nach Klinikaufenthalt auf 48,6 % (*n* = 881) bzw. 14,3 % (*n* = 259). Die logistische Regressionsanalyse zeigt eine signifikant steigende Wahrscheinlichkeit, ein Mangelernährungsrisiko während des Klinikaufenthaltes zu entwickeln, wenn Einweisungsdiagnosen der ICD-10-Gruppen (International Classification of Diseases and Related Health Problems) Krankheiten des Atmungssystems (OR 2,686; 95 %-KI 1,111–4,575) und Nebendiagnosen der Gruppe Osteopathien und Chondropathien (OR 1,892; 95 %-KI 1,149–3,115) vorliegen sowie bei einem höheren BMI (OR 1,108; 95 %-KI 1,038–1,181), positiveren Gewichtsveränderungen 6 Monate vor Krankenhaus (OR 1,055; 95 %-KI 1,017–1,094) und einer steigenden Anzahl an Behandlungstagen (OR 1,048; 95 %-KI 1,029–1,067).

**Diskussion:**

Sowohl die Identifikation einer initialen Mangelernährung als auch die Prävention eines innerklinischen Mangelernährungsrisikos stellt eine große Herausforderung für die Kliniken dar. Beides ist aber gleichermaßen erforderlich.

**Zusatzmaterial online:**

Zusätzliche Informationen sind in der Online-Version dieses Artikels (10.1007/s00391-021-01900-z) enthalten.

## Abstract (en)

**Background:**

Malnutrition is a major challenge in routine clinical practice and is associated with increased mortality.

**Objectives:**

In the research project *Prevention and treatment of malnutrition in geriatric patients in hospital *funded by the Federal Ministry of Education and Research (BMBF), routine data were analyzed. The aim was to uncover the causes of malnutrition risks acquired in hospital.

**Material and methods:**

Anonymized data from nursing home residents with at least a 3-day hospital stay were analyzed. The study included a total of 2058 residents from 19 nursing homes. The malnutrition risk was assessed by the combined MUST/PEMU (Malnutrition Universal Screening Tool/Nursing Measurement of Malnutrition and its Causes) screening and malnutrition by ESPEN (European Society for Clinical Nutrition and Metabolism) criteria.

**Results:**

Of the residents 36.2% (*n* = 744) had an initial risk of malnutrition and 12.7% (*n* = 262) were already malnourished. The proportions increased to 48.6% (*n* = 881) and 14.3% (*n* = 259) at discharge, respectively. The logistic regression analysis showed a significantly increasing probability of developing a malnutrition risk during the hospital stay with the diagnoses diseases of the respiratory system (OR 2.686; CI 95 1.111–4.575), chondropathy and osteopathy (OR 1.892; CI 95 1.149–3.115) and a higher BMI (OR 0.108; CI 95 1.038–1.181), more positive weight changes 6 months before hospital (OR 1.055; CI 95 1.017–1.094) and an increasing hospital stay (OR 1.048; CI 95 1.029–1.067).

**Conclusion:**

The identification of an initial malnutrition and the prevention of developing a malnutrition risk represent major challenges in clinical practise. Both are equally necessary.

## Einführung und Hintergrund

Mangelernährung stellt im klinischen Alltag ein häufig auftretendes Problem dar. So sind nach einer Auswertung von nutritionDay-Daten deutscher Krankenhäuser 37,4 % der eingeschlossenen PatientInnen als manifest mangelernährt einzustufen. Lediglich 11,6 % werden aber tatsächlich auch als mangelernährt erkannt [22]. Vor allem ältere PatientInnen sind überproportional häufig betroffen [1, 4, 12]. Eine Mangelernährung und Verschlechterungen des Ernährungsstatus sind bei Älteren sowohl im Krankenhaus [9, 16] als auch in der Langzeitpflege [8, 21] mit einer erhöhten Mortalität assoziiert. Allerdings werden in diesen Untersuchungen die Veränderungen des Ernährungsstatus und die Entwicklung eines Mangelernährungsrisikos im Verlauf des Klinikaufenthaltes nur sehr selten untersucht, sondern meist zu Beginn der Behandlung festgestellt und anschließend damit zusammenhängende klinische Effekte untersucht. Das Essverhalten und Gründe für eine reduzierte Nahrungsaufnahme im Krankenhaus hingegen wurden durch die jährlich stattfindenden nutritionDays ausführlich analysiert [17]. Aber auch in diesen Erhebungen gibt es keine zwei Messpunkte für etwa das Gewicht, sodass Veränderungen des Ernährungsstatus im Verlauf der Behandlung nicht abbildbar sind.

Im vom Bundesministerium für Bildung und Forschung (BMBF) geförderten Forschungsprojekt *Prävention und Behandlung von Mangelernährung bei geriatrischen Patienten im Krankenhaus* wurden in Zusammenarbeit mit 2 Stuttgarter Kliniken Praxiskonzepte entwickelt, um einer Mangelernährung bei geriatrischen PatientInnen vorzubeugen, diese zu erkennen und zu behandeln [23]. Die Konzepte sollten mithilfe von Routinedaten von PflegeheimbewohnerInnen mit mindestens 3‑tägigem Klinikaufenthalt auf ihre Wirksamkeit getestet werden. Hierfür wurde ein Vorher-nachher-Design mit Interventions- und Kontrollgruppe entwickelt [11]. Bei der Analyse der Daten vor und nach der Konzeptentwicklung (noch nicht veröffentlicht) zeigten sich deutliche Unterschiede beim Ernährungsstatus der Kohorten in den beiden Erhebungszeiträumen. Tatsächlich hatten die PatientInnen im zweiten Erhebungszeitraum signifikant weniger Gewicht vor der Einweisung und anschließend in der Klinik verloren. Aus den wenigen bisher veröffentlichten Daten und der im Projekt gemachten Beobachtung ergibt sich eine erhebliche Forschungslücke. Ziel ist es daher, die Ursachen eines in der Klinik erworbenen Mangelernährungsrisikos zu ermitteln.

## Methodik und Datenbasis

Die Stichprobe umfasst Daten von PflegeheimwohnerInnen mit Klinikaufenthalt in den Jahren 2015 und 2016 sowie im Zeitraum November 2018 bis November 2019. Die routinemäßigen Gewichtserhebungen in den Einrichtungen erlauben so die Betrachtung von Gewichtsverläufen vor und im Zusammenhang mit Klinikaufenthalten.

In die Auswertung flossen Daten von 19 Pflegeeinrichtungen im Raum Stuttgart ein. Die vorliegenden Daten wurden retrospektiv erhoben und erst nach einer gründlichen Anonymisierung in engem Austausch mit den jeweiligen Datenschutzbeauftragten ausgewertet. Einschlusskriterien sind lediglich ein Alter ≥ 65 Jahre und ein mindestens 3‑tägiger Klinikaufenthalt. Die Routinedaten umfassen Gewichts‑, BMI-Verläufe und Mangelernährungsscreenings 6 Monate vor bis unmittelbar nach dem Klinikaufenthalt, Alter, Geschlecht, Pflegegrad, Stürze ggf. Versterben sowie Dauer der Klinikaufenthalte. Den Entlassbriefen wurden Einweisungsdiagnosen sowie Nebendiagnosen entnommen, entsprechend dem ICD-10-Katalog (International Classification of Diseases and Related Health Problems) gruppiert und der Charlson-Komorbiditätsindex in der modifizierten Fassung nach Quan ermittelt [13]. Informationen zu verabreichten Medikamenten, erfolgten Operationen oder intensivmedizinischen Behandlungen liegen nur für eine kleine Gruppe vor und konnten daher nicht berücksichtigt werden.

Gewicht, BMI und Screeningergebnis des Instrumentes Pflegerische Erfassung von Mangelernährung und deren Ursachen in der stationären Langzeit‑/Altenpflege (PEMU) wurden, ausgehend vom Aufnahmedatum, für 6 Monate vorher, 3 Monate vorher, kurz vorher (max. 14 Tage) und unmittelbar nach dem Klinikaufenthalt erfasst. Nach der Entlassung wurden alle BewohnerInnen innerhalb eines Tages in den Einrichtungen gewogen.

Um BewohnerInnen mit Risiko für eine Mangelernährung zu identifizieren, werden die Kriterien Gewichtsverlust und niedriger BMI des validen Screeninginstrumentes Malnutrition Universal Screening Tool (MUST) mit den Ergebnissen des in den Einrichtungen verwendeten Instrumentes PEMU kombiniert ausgewertet [18, 20, 20]. Dieses Vorgehen wird gewählt, da anhand der Daten das MUST-Kriterium Nahrungskarenz nicht erfasst werden kann, in der PEMU-Einschätzung jedoch enthalten ist. Gleichzeitig wird die PEMU in den Einrichtungen nicht so regelmäßig durchgeführt, dass diese Einschätzung ausreichen würde, um BewohnerInnen mit einem initialen Mangelernährungsrisiko sicher zu erfassen. Ist mindestens eine Einschätzung, per PEMU oder MUST unmittelbar vor bzw. nach dem Klinikaufenthalt positiv, wird dies jeweils als Risiko für eine Mangelernährung eingestuft. Zur Identifikation einer manifesten Mangelernährung werden die Konsenskriterien der European Society for Clinical Nutrition and Metabolism (ESPEN) herangezogen und auf alle Fälle angewendet [5]. Allerdings können nur die enthaltenen Kriterien niedriger BMI bzw. altersadaptierter BMI in Kombination mit einem ausgeprägten Gewichtsverlust berücksichtigt werden. Messungen der Körperzusammensetzung, zur Bestimmung eines niedrigen Fettfreie-Masse-Index (FFMI), als weiteres Kriterium einer manifesten Mangelernährung liegen nicht vor (Zusatzmaterial online: Tab. 1). Hauptaugenmerk zur Beschreibung von Veränderungen des Ernährungsstatus im Zuge des Klinikaufenthaltes liegen darum auf einem erheblichen Gewichtsverlust im Zusammenhang mit dem Klinikaufenthalt (≥ 5 %) sowie einem neu aufgetretenen Mangelernährungsrisiko.

**Table 1:** 

Stichprobe *n*	–	2058
Geschlecht, *n* (%)	Männer	763 (37,1)
	Frauen	1295 (62,9)
Alter, MW ± SD	In Jahren	83,53 ± 7,49
Behandlungstage, MW ± SD	–	10,75 ± 9,58
Gewicht vor KH, MW ± SD	In kg	66,45 ± 15,15
BMI vor KH, MW ± SD	In kg/m^2^	24,75 ± 5,06
Einweisungsdiagnosen nach ICD-10-Gruppen, *n* (%)	Verletzungen, Vergiftungen und bestimmte andere Folgen äußerer Ursachen	294 (14,3)
	Krankheiten des Atmungssystems	265 (12,9)
	Krankheiten des Verdauungssystems	260 (12,6)
	Symptome und abnorme klinische Befunde	205 (10,0)
	Krankheiten des Kreislaufsystems	204 (9,9)
	Unbekannt	245 (11,9)
Mortalität im KH, *n* (%)	–	245 (11,9)
Pflegegrad *n* (%)	0	11 (0,5)
	1	65 (3,2)
	2	392 (19,0)
	3	563 (27,4)
	4	608 (29,6)
	5	418 (20,3)
Komorbiditätsindex nach Charlson, MW ± SD	0–29 Punkte	7,16 ± 2,11
Chronische Erkrankungen, *n* (%)	Arterielle Hypertonie	1288 (62,6)
	Demenz	879 (42,7)
	Niereninsuffizienz	641 (31,1)
	Diabetes mellitus	630 (30,6)
	Z. n. Apoplex	417 (20,3)
Ernährungsstatus bei Aufnahme, *n* (%)	Mangelernährungsrisiko (MUST/PEMU)	744 (36,2)
	Manifeste ME (ESPEN)	262 (12,7)
Ernährungsstatus bei Entlassung, *n* (%)	Mangelernährungsrisiko (MUST/PEMU)^a^	881 (48,6)
	Manifeste ME (ESPEN)^a^	259 (14,3)
	Gestellte ME-Diagnosen (Mangelernährung, Kachexie und Sarkopenie)	90 (4,4)

*n* = 1813

*ESPEN* European Society for Clinical Nutrition and Metabolism, *ICD-10* International Classification of Diseases and Related Health Problems, *KH* Krankenhaus, *ME* Mangelernährung, *SD* Standardabweichung, *Z.* *n.* Zustand nach

^a^Ohne in der Klinik Verstorbene

Ursprünglich beinhaltet der Datensatz 2721 Fälle; nach Bereinigung um Fälle ohne Angaben zu Diagnosen verbleiben 2556. Um den Einfluss von Gewichtschwankungen aufgrund des Ausschwemmens von Ödemen zu minimieren, werden in dieser Analyse PatientInnen mit der Nebendiagnose Herzinsuffizienz oder der Aufnahmediagnose dekompensierte Herzinsuffizienz ausgeschlossen, wodurch sich der Datensatz auf 2058 Fälle reduziert. Die Datenanalyse erfolgt mit SPSS 24®. Zur Identifikation von Risikofaktoren für Gewichtsverlust und Entwicklung eines Mangelernährungsrisikos während des Klinikaufenthaltes wird eine logistische Regressionsanalyse angewendet. Die eingeschlossenen Variablen wurden anhand theoretischer Vorüberlegungen ausgewählt, wie etwa höhere Prävalenzraten bei bestimmten chronischen Erkrankungen. Die Modelle wurden um die Faktoren Erhebungszeitraum, behandelnde Klinik, Interventions- und Kontrollgruppe, Charlson-Komorbiditätsindex, hoher Pflegegrad, Alter sowie Geschlecht adjustiert. Der Einschluss der Variablen in das Modell erfolgte gleichzeitig. Fälle mit fehlenden Daten wurden aus der Regression ausgeschlossen. Ein *p* < 0,05 wird als signifikant betrachtet.

Für das gesamte Forschungsprojekt liegt ein positives Ethikvotum des Ethikkomitees der Deutschen Gesellschaft für Pflegewissenschaften in Witten vor (Antrag-Nr. 17-005).

## Ergebnisse

In die Auswertung fließen insgesamt 2058 Fälle ein. Die Stichprobe (Tab. 1) ist entsprechend den Auswahlkriterien betagt (83,5 Jahre; SD ± 7,5) und mit 62,9 % (*n* = 1295) eher weiblich. Mit 49,9 % (*n* = 1016) weist fast die Hälfte einen Pflegegrad von 4 oder 5 auf; der Charlson-Komorbiditätsindex liegt bei 7,2 (SD ± 2,1).

### Ernährungsstatus und Veränderungen

Ein Großteil der PatientInnen verliert bereits vor Klinikeinweisung an Gewicht. Im Zeitraum 6 Monate vor dem Klinikaufenthalt beträgt dieser Verlust im Mittel −1,8 % (SD ± 7,7; *n* = 1166), 3 Monate vor der Aufnahme −1,3 % (SD ± 5,9; 1443) des Körpergewichtes. In den 3 Monaten vor Klinikeinweisung haben 20,1 % (*n* = 288) mindestens 5 % ihres Körpergewichtes verloren, 13,2 % (*n* = 154) mindestens 10 % in den vorhergehenden 6 Monaten. Dementsprechend weisen 36,2 % (*n* = 744) bei der Aufnahme ein Mangelernährungsrisiko und 12,7 % (*n* = 262) eine manifeste Mangelernährung auf. Im Zusammenhang mit Klinikaufenthalten verlieren die PatientInnen im Mittel 1,7 kg (SD ± 4,1 kg) oder 2,3 % (SD ± 6,) ihres Körpergewichtes, 21,9 % (*n* = 396) verlieren ≥ 5 % ihres Körpergewichtes. Der Anteil an PatientInnen mit Mangelernährungsrisiko steigt nach dem Klinikaufenthalt auf 48,6 % (*n* = 881), der der manifesten Mangelernährung auf 14,3 % (*n* = 259).

### Regression

Die logistischen Regressionsmodelle Gewichtsverlust ≥ 5 % (Chi-Quadrat(27) = 97,775, *p* < 0,001, *n* = 859) und neues Mangelernährungsrisiko (Chi-Quadrat(28) = 136,318, *p* < 0,001, *n* = 1012) zeigen jeweils hochsignifikante Risikofaktoren mit starken Effekten (Zusatzmaterial online: Tab. 2 und 3). Das Risiko eines Gewichtsverlustes ≥ 5 % wird signifikant durch Einweisungsdiagnosen der ICD-10-Gruppen endokrine, Ernährungs- und Stoffwechselkrankheiten (OR 2,768; *p* 0,048), Krankheiten des Nervensystems (OR 2,934; *p* 0,030), Krankheiten des Atmungssystems (OR 2,274; *p* 0,0161), Krankheiten des Kreislaufsystems (OR 2,159; *p* 0,033), die Nebendiagnose Demenz (OR 1,490; *p* 0,026) sowie positivere Gewichtsverläufe in den 3 Monaten vor der Einweisung (OR 1,063; *p* 0,010) und mit steigender Anzahl an Behandlungstagen erhöht (OR 1,063; *p* < 0,001). Die Wahrscheinlichkeit, ein Mangelernährungsrisiko während des Klinikaufenthaltes zu entwickeln, steigt signifikant mit den Einweisungsdiagnosen der Gruppe Krankheiten des Atmungssystems (OR 2,156; *p* 0,033), den Nebendiagnosen Osteopathien und Chondropathien (OR 1,892; *p* 0,012) sowie einem höheren BMI (OR 1,108; *p* 0,002), positiveren Gewichtsveränderungen 6 Monate vor dem Krankenhaus (OR 1,055; *p* 0,004) und einer steigenden Anzahl an Behandlungstagen (OR 1,048; *p* < 0,001).

## Diskussion

Sowohl die Literatur als auch die bisherigen Analysen im Projekt zeigen deutlich, dass das Problem Mangelernährung im klinischen Bereich häufig auftritt, aufgrund mangelhaften oder fehlenden Screenings unentdeckt bleibt und sich dann negativ auf das Outcome auswirkt [7, 8, 15, 22]. Dies verdeutlicht die Wichtigkeit, Mangelernährung frühzeitig zu erkennen und dieser, wenn möglich, im Vorfeld bereits entgegenzuwirken. Die Prävention von Mangelernährung in den verschiedenen Versorgungssettings wird vom pflegerischen Expertenstandard *Ernährungsmanagement zur Förderung der oralen Ernährung in der Pflege* als zentrales Ziel aufgegriffen [2, 6]. Wie gut dies aber schließlich gelingt und inwieweit ungewollte Gewichtsverluste oder Verschlechterungen des Ernährungsstatus bei PflegeheimbewohnerInnen Teil eines natürlichen Alterungsprozesses sind, bleibt häufig unklar. Deutlich wird aber, dass ein erheblicher Anteil der PflegeheimbewohnerInnen bereits mit einem Mangelernährungsrisiko in die Klinik aufgenommen wird. Gleichzeitig zeigen Studien, dass eine individualisierte Ernährungstherapie das Outcome wesentlich verbessern kann [19]. So weisen etwa bei Sanson et al. 37,2 % der internistischen Patienten ein hohes Mangelernährungsrisiko auf [15]. Wird dann ernährungsmedizinisch interveniert, kann auch entsprechend eine Mangelernährung kodiert werden [3]. Dies kann aber nur gelingen, wenn die initial bereits Mangelernährten oder Gefährdeten auch erkannt werden. Es kann allerdings nicht davon ausgegangen werden, dass dies aktuell regelhaft geschieht, da in den Diagnoselisten Mangelernährungsdiagnosen (4,4 %; *n* = 90) kaum vorkommen. Wie eingangs erläutert, setzt sich der hier analysierte Datensatz aus 2 Erhebungen zusammen. Für die kleinere Kohorte der zweiten Erhebung (*n* = 604) konnten auch Informationen zu Operationen, Medikamenten bei Entlassung, Aufenthalten auf Intensivstation und den behandelnden Fachabteilungen erfasst werden, was bei der ersten Erhebungsphase leider nicht der Fall war. Hierdurch zeigten sich jedoch keine signifikanten Risikoveränderungen. In einer größeren Kohorte wäre dies jedoch denkbar. Diese potenziellen Risikofaktoren nicht ausreichend beurteilen zu können, stellt eine wesentliche Limitation dieser Datenauswertung dar. Es wurde/wurden außerdem keine Unterscheidung oder getrennte Kalkulationen zwischen den Interventions- und Kontrollgruppen vorgenommen, da der Einfluss der Konzeptentwicklung hier zu vernachlässigen ist. In der untersuchten Gruppe sind im Zeitraum nach der Konzepteinführung lediglich 59 Fälle den Interventionsstationen zuzuordnen. Dennoch wurde in der Regressionsanalyse zumindest dementsprechend adjustiert.

In der hier untersuchten Gesamtgruppe führen auch die konkreten Einweisungsdiagnosen nicht zu einer Veränderung des Risikos, vermutlich da manche Einweisungsdiagnosen relativ selten sind, z. B. Apoplex mit 3 % (*n* = 62), chronische Wunden mit 2 % (*n* = 41), Myokardinfarkte mit 0,7 % (*n* = 15) sowie Lungenembolien mit 0,7 % (*n* = 14). Lediglich die Gruppierung nach ICD-10-Hauptgruppen weist teilweise auf Risikoerhöhungen hin. Interessanterweise ist der Effekt der meisten chronischen Erkrankungen oder der Gesamtmorbiditätsindex auf das Risiko für Gewichtsverluste oder die Entwicklung eines Mangelernährungsrisikos eher gering. Dies überrascht zunächst, lässt sich aber durch die generell ähnlich ausgeprägte Morbidität in der Kohorte erklären. So weisen 69,6 % (*n* = 1433) einen Charlson-Komorbiditätsindex zwischen 5 und 8 Punkten auf.

Inwiefern sich das Gewicht während des Klinikaufenthaltes ändert, wird relativ selten untersucht. Grass et al. erhoben verschiedene Parameter 10 Tage vor und 30 Tage nach einem chirurgischen Eingriff. 46 % verloren dabei mehr als 5 % ihres Körpergewichtes, wobei als signifikante Risikofaktoren ein niedriger BMI zur Baseline, niedriger Oberarmumfang, Eingriffe im oberen Gastrointestinaltrakt, weibliches Geschlecht und ein erhöhtes CRP identifiziert wurden [10]. Die Risikoerhöhung bei Erkrankungen des Atmungssystems deutet hier ebenfalls erhebliche Effekte akut entzündlicher Prozesse an.

In der Studie von Alvarez et al. kam es bei den über 70-Jährigen zu einem signifikanten Gewichtsverlust von −1,7 kg; der Anteil Mangelernährungsrisiko stieg geringfügig von 54,6 auf 57,5 %, während der Anteil manifester Mangelernährung von 25,5 auf 21,3 % sank. Risikofaktoren wurden allerdings keine ermittelt [1]. In einer weiteren Studie kam es dagegen in einer kleineren Kohorten nur zu geringfügigen mittleren Gewichtsveränderungen, jedoch zu einem erheblichen Verlust an fettfreier Masse und damit vermutlich v. a. Muskelmasse. Auch der Phasenwinkel als Zeichen einer schlechten Nährstoffversorgung verschlechterte sich während des Klinikaufenthaltes. Diese Verschlechterung des Ernährungsstatus ist signifikant mit einem höheren Alter und einer bereits bestehenden Mangelernährung zur Baseleine assoziiert [14], was sich in der hier analysierten Kohorte nicht bestätigte.

Nun zeigt sich hier in dieser Untersuchung ein verhältnismäßig ausgeprägter Gewichtsverlust im Zusammenhang mit den Klinikaufenthalten. Dabei ist zu berücksichtigen, dass die Kohorte ausschließlich aus BewohnerInnen von Pflegeeinrichtungen besteht. Damit sind die Ergebnisse nicht allgemein auf geriatrische oder ältere PatientInnen übertragbar. Da PflegeheimbewohnerInnen vermutlich eine vergleichsweise größere Morbidität und Pflegebedürftigkeit aufweisen, wäre eine stärkere Gefährdung für Mangelernährung und ungewollte Gewichtsverluste plausibel. Vergleichbare Erhebungen mit einer solchen Kohorte wurden aber bislang nicht durchgeführt. Der Zusammenhang eines längeren Klinikaufenthaltes mit steigendem Risiko für Gewichtsverluste bzw. Entwicklung eines Mangelernährungsrisikos kommt deutlich zum Vorschein. Unklar bleibt an dieser Stelle, was Ursache und Wirkung ist. Führt der längere Klinikaufenthalt zu zunehmenden Gewichtseinbußen, führen die Gewichtsverluste zu einem längeren Klinikaufenthalt oder ist am ehesten eine Mischung aus beiden Effekten anzunehmen? Im Gegensatz zu der eingangs angedeuteten These wirkt sich in dieser Kohorte ein initial vergleichsweise besserer Ernährungsstatus nicht protektiv auf Gewichtseinbußen während der stationären Behandlung aus. Ein besserer Ausgangsstatus führt eher zu einer Gewichtsabnahme ≥ 5 %. Ein möglicher Erklärungsansatz ist der Umstand, dass eine initiale Mangelernährung mit einem häufigeren Versterben assoziiert ist. Dies hat eine frühere Datenanalyse mit dem T_0_-Datensatz deutlich gemacht [8]. Da in dieser Analyse aber die Gewichtsveränderungen in der Klinik anhand der Gewichtsdifferenz vor/nach dem Klinikaufenthalt errechnet werden, werden hier alle in der Klinik Verstorbenen nicht miterfasst. Dieser Umstand könnte diesen überraschenden Effekt mitverursacht haben. Auch andere eigentlich plausibel erscheinende Risikofaktoren wie Alter, Geschlecht, Morbidität und ausgeprägte Pflegebedürftigkeit spielen hierbei keine signifikante Rolle. Diese überraschenden Befunde sollten ggf. mit einem gezielteren Untersuchungsdesign und größeren Gruppen noch einmal überprüft werden. Eine mögliche Erklärung für den kaum vorhandenen Einfluss von Morbidität oder Pflegebedürftigkeit wäre, dass die Betroffenen erstens schon häufig mit einem reduzierten Ernährungsstatus aufgenommen werden, der sich dann vielleicht nicht mehr wesentlich verschlechtern kann. Zweitens werden diese PatientInnen u. U. verhältnismäßig gut versorgt, da die Pflegekräfte bei angespannter Personallage dazu neigen könnten, sich schwerpunktmäßig um die besonders hilfsbedürftigen PatientInnen zu kümmern. Auch diesen Thesen sollte in weiteren Untersuchungen nachgegangen werden. Die größten Schwierigkeiten oder Limitationen dieser Arbeit gehen auf die Auswertung von Routinedaten zurück. Diese weisen teilweise nichterklärbare Lücken auf. So fehlen z. B. für den zweiten Erhebungszeitraum überdurchschnittlich viele Entlassbriefe, was dazu führte, dass u. a. Einweisungsdiagnosen teilweise nicht vorliegen. Auch problematisch ist, dass nicht immer ein Gewicht vor Krankenhaus vorliegt, da die meisten Krankenhausaufnahmen nicht geplant sind und das letzte gemessene Gewicht dann u. U. zu lange zurückliegt. Gleichzeitig sind manche BewohnerInnen erst in den Monaten vorher in die Langzeitpflege eingezogen, wodurch in diesen Fällen eine Betrachtung der Gewichtsverläufe vor Krankenhauseinweisung nicht möglich war. Diese wird aber benötigt, um das Mangelernährungsrisiko und eine manifeste Mangelernährung einschätzen zu können, da das in der Langzeitpflege verwendete Instrument PEMU relativ selten zur Anwendung kommt. Dadurch wird u. U. die tatsächliche Prävalenz einer Mangelernährung bzw. ein Mangelernährungsrisiko in dieser Kohorte noch unterschätzt.

## Fazit für die Praxis

- Ein hoher Anteil der PatientInnen, die aus einem Pflegeheim aufgenommen werden, weist initial bereits ein Mangelernährungsrisiko oder eine manifeste Mangelernährung auf.
- Es ist von essenzieller Bedeutung, die bereits mit einer Mangelernährung aufgenommenen PatientInnen zu erkennen, um so eine gezielte Ernährungstherapie zu ermöglichen.
- Es ist erforderlich, im Verlauf des Aufenthaltes Mangelernährungsscreenings zu wiederholen, da sich häufig ein Risiko bzw. eine Mangelernährung erst während des Aufenthaltes manifestiert. Das Ernährungsmanagement ist zudem so auszurichten, dass allgemein eine Prävention von Mangelernährung unterstützt wird.

## Supplementary Information




