# Supplementary material for: Weight loss and malnutrition risk in geriatric patients
Source: Z Gerontol Geriatr. 2021 May 5;54(8):789–94. [Article in German] doi: 10.1007/s00391-021-01900-z (PMC8636424; doi:10.1007/s00391-021-01900-z)
Supplement: Supplementary file 1 [file 391_2021_1900_MOESM1_ESM.docx]

Tabelle 1: Risiko für und manifeste Mangelernährung (eigene Darstellung nach [18, 20])

|  | **Messmethode** | **Kriterien** | **Gewichtung (Punkte)** | **Einschätzung** |
| --- | --- | --- | --- | --- |
| Mangelernährungs-risiko | MUST | BMI | ≥ 20 (0)  18,5-20 (1)  ≤18,5 (2) | ≥ 1 Punkt = Risiko |
|  |  | Gewichtsverlust 3-6 Monate | ≤ 5% (0)  5-10% (1)  ≥10% (2) |  |
|  |  | akute Nahrungskarenz | > 5 Tage (2), hier nicht beurteilbar |  |
|  | PEMU | äußerer Eindruck, BMI ≤20, unbeabsichtigter Gewichtsverlust ≥5%/ 1Monat oder ≥10%/ 6 Monate, auffällig geringe Essmenge, erhöhter Bedarf/ Verlust | | mindestens 1 Parameter ja = Risiko |
| Manifeste Mangelernährung | ESPEN Kriterien | BMI oder | < 18,5 | mangelernährt |
|  |  | Gewichtsverlust und | > 10 %, unbestimmte Zeit oder > 5% letzte 3 Monate | mangelernährt |
|  |  | BMI  oder | < 20 wenn < 70 Jahre oder < 22 wenn ≥ 70 Jahre |  |
|  |  | FFMI | < 15 bei Frauen, < 17 bei Männern, hier nicht beurteilbar |  |

*Tabelle 2: Logistische Regression Gewichtsverlust ≥ 5%, N=859 (eigene Darstellung)*

|  | OR | CI (95%) | Wald | p | R² | f |
| --- | --- | --- | --- | --- | --- | --- |
| **Gewichtsverlust ≥ 5% im KH (n=209)** |  |  |  | < 0,001 | 0,160 | 0,436 |
| ED Endokrine, Ernährungs- uns Stoffwechselkrankheiten | 2,768 | 1,011-7,580 | 3,925 | 0,048 |  | |
| ED Krankheiten des Nervensystems | 2,934 | 1,110-7,759 | 4,709 | 0,030 |  | |
| ED Krankheiten des Atmungssystems | 2,274 | 1,165-4,440 | 5,794 | 0,016 |  | |
| ED Krankheiten des Kreislaufsystems | 2,159 | 1,063-4,386 | 4,534 | 0,033 |  | |
| ND Demenz | 1,490 | 1,050-2,114 | 4,982 | 0,026 |  |  |
| Behandlungstage | 1,070 | 1,048-1,092 | 41,602 | < 0,001 |  |  |
| Gewichtsveränderungen 3 Monate bis vor KH in % | 1,063 | 1,015-1,114 | 6,693 | 0,010 |  |  |
| Chi-Quadrat(27) = 97,775, p < 0,001, n = 859 | | | | | | |
| ED = Einweisungsdiagnose; ND = Nebendiagnose; KH = Krankenhaus | | | | | | |
| Nicht signifikant: BMI vor KH; Gewicht vor KH; Gewichtsveränderungen 6 Monate bis vor KH in %; Stürze vor KH; ND Diabetes mellitus; ND Chronische Lungenerkrankungen; ND Chronische Darmerkrankungen; ND Lebererkrankungen; ND Arthropathien, Arthrose und Gicht; ND Osteopathien und Chondropathien; ND Niereninsuffizienz; ND Parkinson; ND Sonstige degenerative Erkrankungen Nervensystem; ED Gi-Erkrankungen und Symptome; ED Neubildungen; ED Krankheiten des Blutes und blutbildender Organe; ED Psychische und Verhaltensstörungen; ED Krankheiten der Haut und Unterhaut; ED Symptome und abnorme klinische und Laborbefunde; ED Verletzungen und Vergiftungen | | | | | | |

*Tabelle 3: Logistische Regression im Krankenhaus erworbenes Mangelernährungsrisiko, N=1012 (eigene Darstellung)*

|  | OR | CI (95%) | Wald | p | R² | f |
| --- | --- | --- | --- | --- | --- | --- |
| **Risiko ME im KH erworben (n=167)** |  |  |  | P < 0,001 | 0,213 | 0,520 |
| ED Krankheiten des Atmungssystems | 2,255 | 1,111-4,575 | 5,072 | 0,024 |  | |
| ND Osteopathien und Chondropathien | 1,892 | 1,149-3,115 | 6,274 | 0,012 |  |  |
| BMI vor KH | 1,108 | 1,038-1,181 | 9,584 | 0,002 |  |  |
| Gewichtsveränderungen 6 Monate bis vor KH in % | 1,055 | 1,017-1,094 | 8,137 | 0,004 |  |  |
| Behandlungstage | 1,048 | 1,029-1,067 | 25,602 | < 0,001 |  |  |
| (Chi-Quadrat(28) = 136,318, p < 0,001, n = 1012) | | | | | | |
| BMI = Body Mass Index; ED = Einweisungsdiagnose; ME = Mangelernährung; ND = Nebendiagnose; KH = Krankenhaus | | | | | | |
| Nicht signifikant: Gewicht vor KH; Gewichtsveränderungen 3 Monate bis vor KH in %; Stürze vor KH; ND Diabetes mellitus; ND Demenz; ND Chronische Lungenerkrankungen; ND Chronische Darmerkrankungen; ND Lebererkrankungen; ND Arthropathien, Arthrose und Gicht; ND Niereninsuffizienz; ND Parkinson; ND Sonstige degenerative Erkrankungen Nervensystem; ED GI-Erkrankungen und Symptome; ED Neubildungen; ED Krankheiten des Blutes und blutbildender Organe; ED Endokrine, Ernährungs- und Stoffwechselerkrankungen; ED Psychische und Verhaltensstörungen; ED Erkrankungen des Nervensystems; ED Krankheiten des Kreislaufsystems; ED Krankheiten der Haut und Unterhaut; ED Symptome und abnorme klinische und Laborbefunde; ED Verletzungen und Vergiftungen | | | | | | |
